# Trajectories of illness uncertainty among parents of children with atypical genital appearance due to differences of sex development

**DOI:** 10.1093/jpepsy/jsae043

**Published:** 2024-06-10

**Authors:** Katherine A Traino, Lucia M Ciciolla, Megan N Perez, John M Chaney, Ginger Welch, Laurence S Baskin, Cindy L Buchanan, Yee-Ming Chan, Earl Y Cheng, Douglas E Coplen, Amy B Wisniewski, Larry L Mullins

**Affiliations:** Department of Psychology, Center for Pediatric Psychology, Oklahoma State University, Stillwater, OK, United States; Department of Psychology, Center for Pediatric Psychology, Oklahoma State University, Stillwater, OK, United States; Division of Oncology, The Children's Hospital of Philadelphia, Philadelphia, PA, United States; Department of Psychology, Center for Pediatric Psychology, Oklahoma State University, Stillwater, OK, United States; Department of Human Development and Family Sciences, Oklahoma State University, Stillwater, OK, United States; Department of Urology, University of California San Francisco Medical Center, San Francisco, CA, United States; Department of Psychiatry, University of Colorado Anschutz Medical Campus, Aurora, CO, United States; Division of Endocrinology, and Harvard Medical School, Boston Children’s Hospital, Boston, MA, United States; Department of Urology, Ann & Robert H. Lurie Children’s Hospital of Chicago, Chicago, IL, United States; Division of Urologic Surgery, St. Louis Children’s Hospital, St Louis, MO, United States; Department of Psychology, Center for Pediatric Psychology, Oklahoma State University, Stillwater, OK, United States; Department of Psychology, Center for Pediatric Psychology, Oklahoma State University, Stillwater, OK, United States

**Keywords:** differences of sex development, variations in sex characteristics, variations in sex traits, parent coping, illness uncertainty

## Abstract

**Objective:**

The present study aimed to identify distinct trajectories of parental illness uncertainty among parents of children born with atypical genital appearance due to a difference of sex development over the first year following diagnosis. It was hypothesized that four trajectory classes would emerge, including “low stable,” “high stable,” “decreasing,” and “increasing” classes, and that select demographic, familial, and medical factors would predict these classes.

**Methods:**

Participants included 56 mothers and 43 fathers of 57 children born with moderate to severe genital atypia. Participants were recruited from eleven specialty clinics across the U.S. Growth mixture modeling (GMM) approaches, controlling for parent dyad clustering, were conducted to examine classes of parental illness uncertainty ratings over time.

**Results:**

A three-class GMM was identified as the best-fitting model. The three classes were interpreted as “moderate stable” (56.8%), “low stable” (33.0%), and “declining” (10.3%). Findings suggest possible diagnostic differences across trajectories.

**Conclusions:**

Findings highlight the nature of parents’ perceptions of ambiguity and uncertainty about their child’s diagnosis and treatment the year following their child’s birth/diagnosis. Future research is needed to better understand how these trajectories might shift over the course of the child’s development. Results support the development of tailored, evidence-based interventions to address coping with uncertainty among families raising a child with chronic health needs.

Atypical genital appearance at birth is often a result of a disorder/difference of sex development (DSD). DSDs are congenital conditions in which there is a discrepancy between a person’s chromosomes, gonads, and/or phenotypic sex (Achermann & Hughes, 2011). Approximately 0.2% of children are born with an atypical genital appearance, with a subset being due to a DSD condition (Lee et al., 2016). Overall, DSD prevalence (with and without atypical genital appearance) ranges from 0.018% to 1.7% of live births in the United States (Sandberg & Gardner, 2022; Sax, 2002). Many DSDs are not life-threatening, but some may pose additional medical risk such as salt-wasting crisis (i.e., the body’s inability to retain sodium) or organ malformations (Wisniewski et al., 2012).

Receiving a work-up for a DSD diagnosis may involve: identifying any possible life-threatening conditions (e.g., salt-wasting crisis, problems with organ development), conducting various genetic and medical tests (e.g., blood tests, physical examinations) to determine the underlying cause of the DSD, if possible, and defining the specific reproductive anatomy to inform any necessary treatment plans and gender-of-rearing decisions (Wisniewski et al., 2012). Receiving a diagnosis varies across types of DSDs. For example, most individuals with 46, XX DSD receive a congenital adrenal hyperplasia diagnosis on newborn screening or other newborn evaluation, whereas only half of individuals with 46, XY ever receive a diagnosis (ALOmran & ALJurayyan, 2021).

Parents of children with a DSD often navigate various decisions, such as selecting a gender of rearing, medical interventions (e.g., glucocorticoid medication), and electing (or not) to conduct functional and/or cosmetic genital surgeries (Wisniewski et al., 2012). Given the limited data on long-term outcomes, such decisions and treatments involve a great deal of uncertainty and require ongoing conversations with providers throughout a child’s development (Cools et al., 2018; Crissman et al., 2011; Duguid et al., 2007; Graziano & Fallat, 2016; Lampalzer et al., 2020). *Illness uncertainty* captures this experience of doubt or loss of control secondary to unpredictable illness-related events or information (Mishel, 1981). DSD-specific factors contributing to illness uncertainty may include limited understanding of their child’s diagnosis or treatment; unpredictability of their child’s health and course of illness and/or treatment; or difficulty understanding the medical information presented.

Parents have qualitatively reported uncertainty as a significant stressor (Crissman et al., 2011). Survey data corroborate these findings, with a subset of parents reporting significant illness uncertainty (Perez et al., 2019; Suorsa et al., 2015). Further, illness uncertainty has been linked to short- (Delozier et al., 2019; Pasterski et al., 2014) and long-term parent adjustment difficulties (Roberts et al., 2020). How illness uncertainty changes over time is unclear and poorly understood in this population. Some findings demonstrate that illness uncertainty does not change (Wolfe-Christensen et al., 2017), whereas other findings show an overall average decrease in illness uncertainty over time (Roberts et al., 2020). Additional exploratory findings demonstrate possible nuanced subgroup differences, such that parents of boys (based on child gender of rearing) demonstrate an increase in illness uncertainty over time, whereas parents of girls showed a decrease (Wolfe-Christensen et al., 2017). While these findings take an important preliminary step in examining how illness uncertainty changes over time, unique subgroup trajectories have yet to be defined.

In sum, illness uncertainty is a salient experience for parents of children with a DSD and is associated with adjustment difficulties (Sandberg & Gardner, 2022). However, despite calls to examine the longitudinal stability and change in illness uncertainty over time (Wright et al., 2009), this remains a gap in pediatric health research. The present study aims to use person-centered statistical approaches [i.e., latent class growth analysis (LCGA) and growth mixture modeling (GMM)] to examine changes in illness uncertainty from baseline to 6- and 12-month follow-up among parents of children born with atypical genital appearance due to a DSD. Based on previous research, it is predicted that there will be four trajectory classes of uncertainty: (a) a stable low class, (b) a stable high class, (c) a decreasing class, and (d) an increasing class. The study also examines possible predictors of class membership, including sociodemographic and family characteristics, diagnostic and medical characteristics, and parental adjustment (i.e., depressive and anxious symptoms). Previous research in DSD has found that parent gender was predictive of classes of depressive symptoms over time, with fathers demonstrating greater improvement in symptoms over time (Perez et al., 2021). Similarly, parent gender is hypothesized to predict class membership, such that mothers will be more likely to be in a “stable high” class. Further, similar to previous research (Wolfe-Christensen et al., 2017), child gender of rearing is expected to predict these classes, with girl gender of rearing predictive of the decreasing class and boy gender of rearing predictive of the increasing class.

## Methods

### Participants and procedures

Participants included 99 parents (56 mothers, 43 fathers) of 57 children diagnosed with moderate to severe genital atypia due to a DSD. The present study used a subset of data collected as part of a larger multisite longitudinal study examining parent adjustment following their child’s DSD diagnosis. Parents were recruited across eleven sites in the U.S. from September 2013 to November 2017. Institutional Review Board approval was obtained at each site prior to consent. Parent eligibility included: (a) being at least 18 years old, (b) English or Spanish fluency, and (c) not having a severe psychiatric disorder. Additionally, their child must: (a) be diagnosed with a DSD with moderate to severe genital atypia, as defined by a Prader score of 3 to 5 for XX DSD and a Quigley score of 3 to 6 for those with a Y chromosome; (b) be less than 2 years of age; (c) not have received genitoplasty at time of recruitment; and (d) not have any organ malformations outside the reproductive system. Study coordinators obtained informed consent, and parents completed questionnaires in-person or at home for baseline, 6 months, and 12 months post-surgery (or post-baseline for parents who opted against surgery for their child). Participants were compensated for participation travel costs.

### Materials

#### Demographic form

Baseline demographic information included child age, parent age, race/ethnicity (i.e., Black/African American, White/Caucasian, Asian/Pacific Islander, Native Hawaiian/Native American, Multi-Racial, and other), education, income, relation to child, family history of DSD diagnoses, and whether parents previously had children. Medical chart review yielded diagnosis, karyotype, gender of rearing, and whether surgery was conducted.

#### Parent Perception of Uncertainty Scale

The Parent Perception of Uncertainty Scale (PPUS) (Mishel, 1983) is a 31-item self-report measure examining parental perceptions of uncertainty regarding their child’s diagnosis (e.g., “I’m certain they will not find anything else wrong with my child.”). Responses are provided on a 5-point Likert-scale (1 = “strongly agree” to 5 = “strongly disagree”) and summed to generate a total score. Higher scores reflect greater illness uncertainty. See supplementary materials for PPUS items. Of note, there are no formal clinical cutoffs for this measure. Additionally, the PPUS is the only validated measure of parental illness uncertainty and has been used across pediatric illness populations. Internal consistency was acceptable (α_baseline_ = 0.92; α_6 months_ = 0.91; α_12 months_ = 0.89).

#### Beck Depression Inventory-II

The Beck Depression Inventory-II (BDI-II) (Beck et al., 1996) is a widely accepted measure of adult symptomatology and contains 21 items measuring self-reported depressive symptoms. Responses are rated on a 4-point Likert-scale and summed to generate total score. Higher scores indicate greater depressive symptoms. Clinical score classifications are as follows: minimal (0–13), mild (14–19), moderate (20–28), and severe anxiety (29–63). Internal consistency was acceptable (α_baseline_ = 0.94).

#### Beck Anxiety Inventory

The Beck Anxiety Inventory (BAI) (Beck et al., 1988) is a widely accepted measure of adult symptomatology and contains 21 items measuring self-reported anxious symptoms. Responses are rated on a 4-point Likert-scale and summed to generate a total score. Higher scores indicate greater anxious symptoms. Clinical score classifications are as follows: minimal (0–7), mild (8–15), moderate (16–25), and severe anxiety (30–63). Internal consistency was acceptable (α_baseline_ = 0.99).

### Statistical analysis

Because this was a secondary analysis of an existing dataset, the current sample was already established and post-hoc power analyses were not conducted (Wolf et al., 2013). For 119 parents over all three timepoints, 59 parents had data for all three timepoints, 40 had data for two timepoints, and 20 had data for one timepoint. Note, only participants with data for at least two timepoints (*N *= 99) were included in the analyses, as the present study focus is on longitudinal examination of data. As several marginalized racial/ethnic identity categories contained one to few participants, these categories were collapsed into White and Black, Indigenous, and People of Color (BIPOC) categories for data analysis. Missing data analyses assessing for associated demographic factors were conducted for each timepoint for all 119 participants. No significant differences were noted at baseline. Consistent with a previously published subset of the present data (Roberts et al., 2020), lower household income was associated with missing data at 6-month follow-up, χ^2^(12, 115) = 28.58, *p* = .005. At 12-month follow-up, BIPOC parents were more likely to have missing data, χ^2^(1, 107) = 5.10, *p* = .024. Pearson and point biserial correlations were conducted to examine relationships between study variables. A one-way ANOVA was conducted to examine the relationship between child gender of rearing and illness uncertainty time points. Descriptive and missingness analyses were conducted in SPSS Version 29. All model analyses were conducted in MPlus (version 8.3; Muthén & Muthén, 2017). Full-information maximum likelihood estimation was used to accommodate missing data, which estimates parameters using all available information and is preferable for smaller sample sizes (Berlin, Parra, et al., 2014; Preacher et al., 2008). Covariates associated with missingness (i.e., income and BIPOC identities) were accounted for using auxiliary functions for latent growth curve modeling (LGCM) and using direct inclusion in LCGA and GMM, as MPlus auxiliary functions are not available in mixture models.

GMM analysis was conducted to examine parent illness uncertainty appraisals across the baseline, 6-month, and 12-month timepoints. GMM is a subtype of latent growth modeling used to assess individual changes over time, and it identifies unobserved, latent, classes of participants with comparable trajectories (Berlin, Parra, et al., 2014). Recommendations propose a four-stepped latent variable mixture modeling, including: problem definition using LGCM, model specification using LCGA, model estimation using GMM, and model selection and interpretation (Berlin, Parra, et al., 2014; Ram & Grimm, 2009). Additionally, class differences were examined.

### LGCM

Hypotheses about growth patterns were generated from theory, existing literature, and preliminary descriptive analyses. To account for non-independent, dyadic observations across mothers and fathers, standard errors were adjusted using complex analyses in the model (TYPE=COMPLEX syntax in Mplus, Muthén & Muthén, 2017), which utilizes the child variable for clustering. Then, LGCM examined the optimal single-group representation of the data over time (Ram & Grimm, 2009) based on intercept only, linear, and quadratic growth curve models.

### LCGA

A model-building approach evaluated the optimal number of latent classes. Previous research was used to generate hypotheses about the expected number of classes and class differences. LCGA was conducted with the intercept and slope variances fixed at zero within classes and with between-class variance free to vary (Berlin, Parra, et al., 2014; Jung & Wickrama, 2008). Fixing within-class variance decreases the number of estimated parameters, which can support convergence for smaller samples (Berlin, Parra, et al., 2014). Due to the small sample size of this rare medical population, this approach was used first. Analyses were conducted to at least one class greater than the 4 hypothesized classes.

### GMM

Using the best-fitting LCGA model, GMM systematically allowed within-class variances to be freely estimated. All model analyses were with maximum likelihood estimation with robust standard errors and chi-square test statistic (MLR) to account for skewness and nonindependence in the data (Muthén & Muthén, 2017). Starting values were initially set and then systematically increased to optimize replication and ensure global maxima rather than local maxima.

### Model selection and interpretation

The optimal number of latent classes was assessed using several information criteria indices, as previously described. Recommended statistics were used to assess goodness of fit for the non-mixture LGCM model. Excellent fit is determined according to the following fit statistics: comparative fit index (CFI) >0.95, root mean square error approximation (RMSEA) <0.05, and standardized root mean residual (SRMR) <0.05 (Berlin, Williams, et al., 2014; Hu & Bentler, 1999). Additionally, lower Bayesian information criteria (BIC) and Akaike information criteria (AIC) indicate a better model fit (Berlin, Williams, et al., 2014; Geiser, 2013; Jung & Wickrama, 2008).

Additional fit indices for mixture modeling approaches (i.e., LCGA and GMM) are recommended. The Lo, Mendell, and Rubin test (LMR) evaluates the model fit by comparing a model with G classes to a model with G-1 classes (Geiser, 2013). A significant LMR suggests model G is the better model (Geiser, 2013). Although the bootstrap likelihood ratio test (BLRT) is considered a better indicator of model fit than LMR, the BLRT is not provided when TYPE=COMPLEX syntax is used in model specification. Therefore, BIC is recommended for model comparison (Berlin, Parra, et al., 2014). Entropy was used to inform the accuracy of participant placement into a class, with values closest to 1 representing better accuracy (Berlin, Williams, et al., 2014; Geiser, 2013; Jung & Wickrama, 2008). No less than 1% of the sample in each class of the final model was accepted (Jung & Wickrama, 2008). Lastly, prior research, theory, and model fit information informed the best-fitting model (Berlin, Parra, et al., 2014).

### Class differences

The R3STEP procedure assesses for covariates without changing class structure (Asparouhov & Muthén, 2014) and was conducted to examine significant (*p* < .05) class differences based on parent demographic factors, including parent relation to child (coded as mother = 0, father = 1), age, child gender of rearing, DSD diagnosis (coded as CAH = 0, other = 1), whether diagnosis was known (coded as known = 0, unknown = 1), whether parents already had children (coded as previous children = 0, no previous children = 1), family DSD history (coded as no family history = 0, family history = 1), and co-occurring anxious and depressive symptoms. Of note, as race/ethnicity and income were associated with missingness, those covariates were included directly in the model. Additionally, most families opted for surgical intervention (*n *=* *52; 91.2%); therefore, sample sizes are too small to examine its association to class membership.

Multiple imputation methods were used to address missingness in predictor variables, including parent age (*n *=* *5), family DSD history (*n *=* *7), anxious symptoms (*n *=* *2), and depressive symptoms (*n *=* *1). Related predictors were clustered into different models (i.e., sociodemographic, child, family, and parent adjustment clusters) and ran as individual cluster sets before including all predictors in a final multivariate model.

## Results

Overall, the observed means and variances for illness uncertainty at each timepoint were as follows: *M*_baseline_ = 64.83, Var_baseline_ = 269.86; *M*_6 months_ = 59.74, Var_6 months_ = 222.73; *M*_12 months_ = 54.60, Var_12 months_ = 165.55. See Table 1 for participant information. See Table 2 for study variable correlations. One-way ANOVA for child gender of rearing and illness uncertainty across time points was not significant, *F*_baseline_(2, 94) = 1.22, *p* = .299, *F*_6 months_(2, 82) = 1.55, *p* = .218, *F*_12 months_(2, 72) = 0.64, *p* = .529.

**Table 1.** Descriptive characteristics.

	Children (*N *=* *57)	Total parents (*N *=* *99)	Mothers (*n *=* *56, 56.6%)	Fathers (*n *=* *43, 43.4%)
Family income, *n* (%)				
$0–19,999	–	10 (10.1%)	9 (16.1%)	1 (2.3%)
$20,000–39,999	–	16 (16.2%)	10 (17.9%)	6 (14.0%)
$40,000–59,999	–	12 (12.1%)	6 (10.7%)	6 (14.0%)
$60,000–79,999	–	10 (10.1%)	5 (8.93%)	5 (11.6%)
$80,000–$99,999	–	13 (13.1%)	7 (12.5%)	6 (14.0%)
$100,000+	–	38 (38.4%)	19 (33.9%)	19 (44.2%)
Parent education, *n* (%)				
High school diploma/GED or less	–	39 (39.4%)	25 (44.6%)	14 (32.6%)
Associate’s/bachelor’s degree	–	37 (37.4%)	19 (33.9%)	18 (41.9%)
Graduate school	–	23 (23.2%)	12 (21.4%)	11 (25.6%)
Parent race, *n* (%)				
White/Caucasian		73 (73.7%)	42 (75.0%)	31 (72.1%)
Black/African American		6 (6.1%)	3 (5.4%)	3 (7.0%)
Asian/Pacific Islander		5 (5.1%)	2 (3.6%)	3 (7.0%)
Native Hawaiian/Native American		0 (0.0%)	0 (0.0%)	0 (0.0%)
Multi-Racial		5 (5.1%)	4 (7.1%)	1 (2.3%)
Others		10 (10.1%)	5 (8.9%)	5 (11.6%)
Parent race—coded, *n* (%)				
White	–	73 (73.7%)	42 (75%)	31 (72.1%)
BIPOC	–	26 (26.3%)	14 (25%)	12 (27.9%)
Parent age at baseline in years^a^ (*M* ± *SD*)	–	32.64 ± 6.46	31.00 ± 5.93	34.76 ± 6.57
Child age at baseline in months^b^ (*M* ± *SD*)	8.86 ± 6.74	–	–	–
Karyotype, *n* (%)				
46, XY	20 (35.1%)	–	–	–
46, XX	33 (57.9%)	–	–	–
Other	4 (7.0%)	–	–	–
DSD diagnosis, *n* (%)				
Congenital adrenal hyperplasia	31 (54.4%)	–	–	–
Other DSD (e.g., 5-alpha reductase deficiency, androgen insensitivity syndrome)	26 (45.6%)	–	–	–
Diagnosis unknown, *n* (%)	14 (24.6%)	–	–	–
Gender of rearing, *n* (%)				
Girl	35 (61.4%)	–	–	–
Boy	20 (35.1%)	–	–	–
Undecided	2 (3.5%)	–	–	–
Surgery, *n* (%)	52 (91.2%)			
Has previous children, *n* (%)	–	44 (44.4%)	25 (44.6%)	19 (44.2%)
Family history of DSD, *n* (%)	–	12 (12.1%)	6 (10.7%)	6 (14.0%)

*Note*. There are 99 parents of 55 children included in the study. BIPOC = Black, Indigenous, and People of Color; DSD = difference of sex development.

a Age missing for five parents (three mothers and two fathers) and one child.

b Family history missing for seven parents (four mothers and three fathers).

**Table 2.** Study variable correlation matrix.

	1. Baseline PPUS	2. 6-Months PPUS	3. 12-Months PPUS	4. BAI	5. BDI-II	6. Relation to child	7. Age	8. Race	9. Income	10. DSD diagnosis	11. Known diagnosis	12. Previous children	13. Family DSD history
*M*	65.25	60.39	54.18	8.37	8.85	–	32.64	–	–	–	–	–	–
*SD*	15.93	15.65	12.63	8.96	9.42	–	6.46	–	–	–	–	–	–
1.	–	0.354**	0.499**	0.225*	0.268**	−0.028	0.101	−0.047	−0.023	0.218*	0.114	0.174	0.043
2.	0.354**	–	0.664**	0.199	0.344**	−0.074	−0.034	0.041	−0.140	0.056	0.126	0.112	−0.062
3.	0.499**	0.664**	–	0.070	0.147	0.057	0.129	−0.090	0.170	−0.122	−0.076	0.140	−0.011

*Note.* BAI = Beck Anxiety Inventory; BDI-II = Beck Depression Inventory-II; DSD = difference of sex development; PPUS = Parent Perception of Uncertainty Scale.

* *p* < .05.

** *p* < .01.

### LGCM

Intercept model was run with auxiliary covariates (i.e., income and BIPOC variables). For the linear model, results indicated a negative residual for variable “s” which was then set to zero. This residual was also set to zero for the quadratic model. In evaluating LGCM fit statistics for both intercept only and linear models, the linear model demonstrated excellent fit for CFI, TLI, RMSEA, and SRMR, and lower AIC, BIC, and SSA-BIC relative to the intercept and quadratic models (see Table 3). Thus, the linear model represents a meaningful non-curvilinear change over time that can be probed further.

**Table 3.** Loglikelihood, information criteria, and entropy tests for LGCM, LCGA, and GMM.

Measure	Intercept	Linear	Quadratic	1 class	2 classes	3 classes	4 classes	5 classes
LGCM								
CFI	0.454	1.000	1.000					
TLI	0.590	1.000	1.000					
RMSEA	0.258	0.000	0.000					
SRMR	0.281	0.093	0.000					
χ^2^	30.408	2.468	0.000					
df	4	3	0					
χ^2^/df	7.602	0.823	0.000					
***φ***	0.554	0.158	0.000					
AIC	2,768.271	2,742.331	2,745.863					
BIC	2,809.793	2,786.448	2,797.766					
SSA-BIC	2,759.264	2,732.761	2,734.605					
LCGA								
Loglikelihood				−1,056.593	−1,034.892	−1,025.142	−1,018.761	−1,012.416
AIC				2,131.185	2,093.783	2,080.283	2,073.521	2,066.832
BIC				2,154.541	2,124.924	2,119.210	2,120.233	2,121.329
SSA-BIC				2,126.119	2,087.028	2,071.893	2,063.388	2,055.010
Entropy				–	0.695	0.764	0.729	0.787
LMR test				–	40.467	18.181	11.899	11.831
LMR, *p*-value				–	0.1980	0.0995	0.5044	0.1274
GMM								
Loglikelihood				−1,029.672	−1,026.309	−1,015.413	−1,009.903	–
AIC				2,079.345	2,078.618	2,064.826	2,059.807	–
BIC				2,105.296	2,112.355	2,108.943	2,111.709	–
SSA-BIC				2,073.715	2,071.300	2,055.256	2,048.548	–
Entropy				–	0.663	0.691	0.743	–
LMR test				–	57.442	19.086	10.273	–
LMR, *p*-value				–	0.0329	0.7334	0.0319	–

*Note*. AIC = Akaike information criteria; BIC = Bayesian information criteria; GMM = growth mixture modeling; LCGA = latent class growth analysis; LGCM = latent growth curve modeling; LMR = Lo, Mendell, and Rubin test; SSA BIC = sample size adjusted Bayesian information criteria.

### LCGA

LCGA was conducted, fixing intercept and slope within class variances to zero. Analyses estimated up to one class greater than the four hypothesized (i.e., five classes; Table 3). Although the four-class model demonstrated some improvement in fit (e.g., decreased AIC and SSA-BIC), the three-class model demonstrated overall optimal fit and a significant improvement from the two-class model. Therefore, a three-class model was indeed the optimal fit.

### GMM

Due to observed variability within classes (see Supplementary Figure), it is likely that constraining within-class variance within the LCGA is not the most appropriate model to represent the data (Berlin, Parra, et al., 2014). Therefore, a series of GMMs were conducted using an iterative, stepwise approach to examine fit when allowing model parameters to vary. Up to four classes were estimated based on the previous LCGA models, systematically allowing the within-class intercepts, slopes, and variances to vary freely with increased starts included as needed until the best loglikelihood was replicated for all models. The final two- and three-class models demonstrated comparably good fit, warranting further comparison (see “Model selection and interpretation” for explanation).

In arriving at the two- and three-class models, the following sequence of analyses was conducted for one-, two-, three-, and four-class models in which all parameters (i.e., intercept and slope means and variances) were initially allowed to freely vary. However, negative slope variances were identified and therefore the best loglikelihood was not replicated in any of the models. Based on these results, slope variances were set to zero. For the one-class model, these were the final parameters (i.e., slope variance set to zero and intercept and slope means and intercept variance allowed to vary freely) that acceptably replicated the best loglikelihood.

When applying these parameters to the two-class model, a negative variance was identified for the second class at the 12-month timepoint. Therefore, the variance for the 12-month timepoint in the second class was defined as a small positive value close to zero (i.e., 0.00001), thus replicating the best loglikelihood. For the two-class model, random starts were increased to confirm best loglikelihood was replicated. This same set of parameters were then applied to the subsequent three-class model. Initially, the best loglikelihood was not replicated, however, random starts were increased and loglikelihood was then successfully replicated and repeated for a second set of increased starts. Parameters were then applied to the four-class model. Results indicated the need to both define the variance for the first class to a small positive value close to zero, as well as optimize the starting values using the OPTSEED function. Then, the best loglikelihood was replicated. See Table 3 for all model results.

### Model selection and interpretation

**Figure 1.**
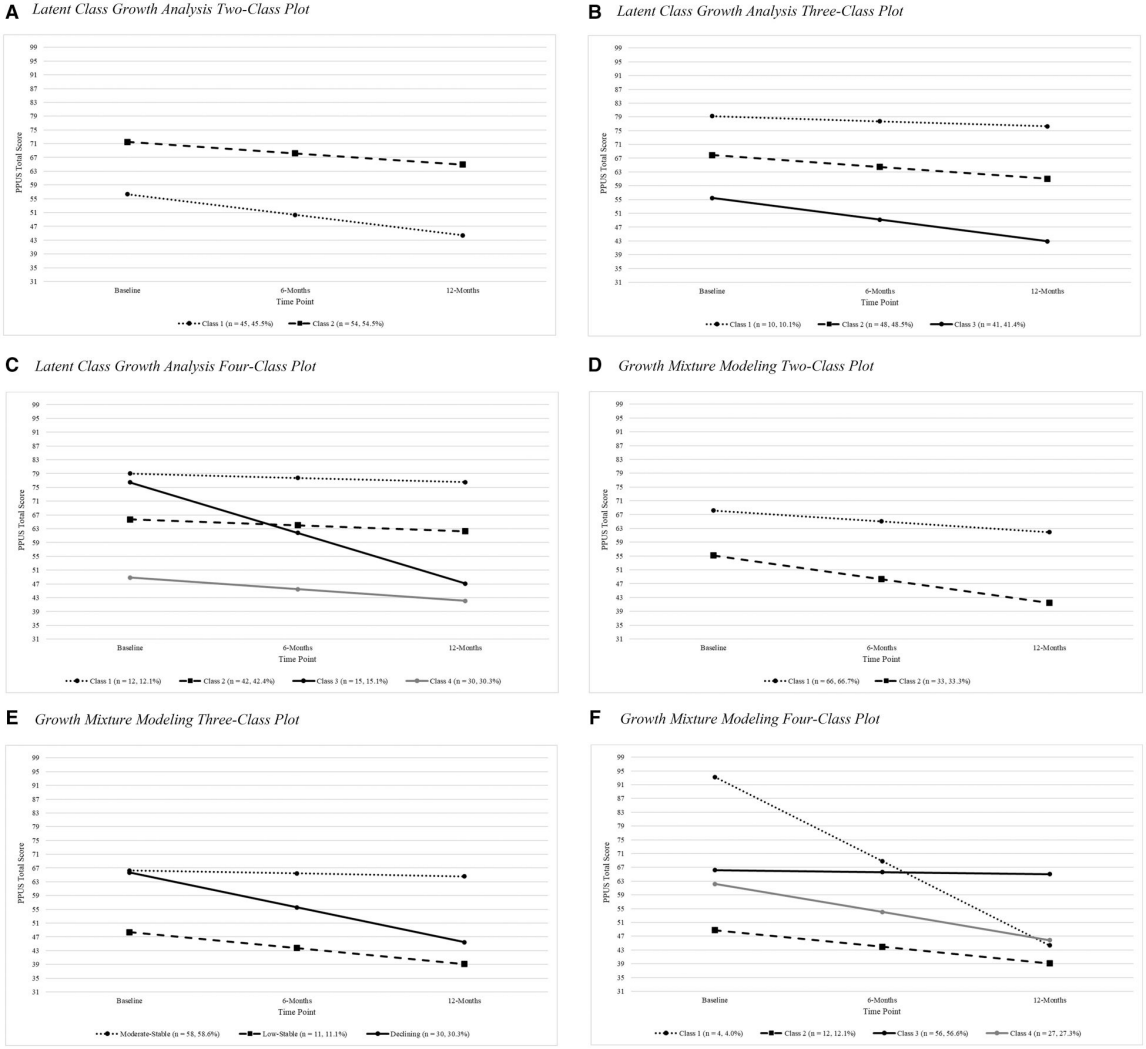
Model selection plots. (A) Latent class growth analysis two-class plot. (B) Latent class growth analysis three-class plot. (C) Latent class growth analysis four-class plot. (D) Growth mixture modeling two-class plot. (E) Growth mixture modeling three-class plot. (F) Growth mixture modeling four-class plot.

Comparison of LCGA and GMM fit statistics demonstrated better model fit for GMM with freely estimated intercept variances, evidenced by comparable loglikelihood, and lower AIC, BIC, and SSA-BIC (Table 3). Analyses of GMM classes demonstrated optimal fit for the three-class model (i.e., greater loglikelihood relative to the two-class model, overall lower BIC). Estimated and sample mean plots for the LCGA two- through four-class and GMM two- through four-class models are included (Figure 1).

### Identified latent classes

For each of the classes in the GMM three-class model, means and variances for each timepoint are shown in Table 4. The three classes were classified as: “moderate-stable,” “low-stable,” and “declining” uncertainty.

**Table 4.** Individual class estimated means at each timepoint.

	Baseline *M*	6 months *M*	12 months *M*
Moderate stable (*n *=* *58, 58.6%)	66.30	65.45	64.60
Low stable (*n *=* *11, 11.1%)	48.39	43.76	39.13
Declining (*n *=* *30, 30.3%)	65.69	55.56	45.43

Parental perception of uncertainty scale					
	1 Strongly disagree	2 Disagree	3 Undecided	4 Agree	5 Strongly agree
1. I do not know what is wrong with my child.					
2. I have a lot of questions without answers.					
3. I am unsure if my child's illness is getting better or worse.					
4. It is unclear how bad my child's physical discomfort will be.					
5. The explanations they give about my child seem hazy to me.					
6. The purpose of each treatment for my child is clear to me.					
7. I do not know when to expect things will be done to my child.					
8. My child's symptoms continue to change unpredictably.					
9. I understand everything explained to me.					
10. The doctors say things to me that could have many meanings.					
11. I can predict how long my child's illness will last.					
12. My child's treatment is too complex to figure out.					
13. It is difficult to know if the treatments and medications my child is getting are helping.					
14. There are so many types of medical staff, it is unclear who is responsible for what.					
15. Because of the unpredictability of my child's illness, I cannot not plan for the future.					
16. The course of my child's illness keeps changing. He/she has good and bad days.					
17. It is vague to me how I will manage the care of my child after leaving the hospital/doctor's office.					
18. It is not clear what is going to happen to my child.					
19. I usually know if my child is going to have a good or bad day.					
20. The results of my child's tests are inconsistent.					
21. The effectiveness of the treatment of my child's illness undetermined.					
22. It is difficult to determine how long it will be before I can care for my child's illness by myself.					
23. I can generally predict the course of my child's illness.					
24. Because of the treatment, what my child can do and cannot do keeps changing.					
25. I am certain they will not find anything else wrong with my child.					
26. They have not given my child a specific diagnosis.					
27. My child's physical distress is predictable; I know when it is going to get better or worse.					
28. My child's diagnosis is definite and will not change.					
29. I can depend on the nurses to be there when I need them.					
30. The seriousness of my child's illness has been determined.					
31. The doctors and nurses use everyday language so I can understand what they are saying.					

Mishel (1983).

#### Moderate-stable uncertainty

The largest class (#1) included 58.6% (*n *=* *58) of parents. Parents in this class initially reported relatively moderate levels of uncertainty (*M*_intercept_ = 70.25, *p* < .001; Var_intercept_ = 75.61, *p* = .044), with levels remaining fairly stable over time (slope = −5.99, *p* = .365). Although there are not any formal clinical cutoffs for uncertainty, reported levels of uncertainty within this class appear moderate compared to the present sample and similar pediatric populations (Mishel, 1983; Perez et al., 2019). Therefore, this class was named “moderate stable.”

#### Declining uncertainty

The next largest class (#3) included 30.3% (*n *=* *30) of parents. Parents in this class initially rated relatively moderate levels of uncertainty at baseline (*M*_intercept_ = 69.42, *p* < .001; Var_intercept_ = 51.31), with lower levels at 6 months (*M*_intercept_ = 54.97), and 12 months (*M*_intercept_ = 44.81; slope = −24.38, *p* = .001). Given that ratings of uncertainty in this class were initially the comparable to the moderate-stable class but by 12 months decreased to levels comparable to the class with the lowest uncertainty ratings (i.e., class #2), this class was labeled the “declining” class.

#### Low-stable uncertainty

The final class (#2) included 11.1% (*n *=* *11) of the sample. Parents in this class initially rated relatively low levels of uncertainty at baseline (*M*_intercept_ = 52.04, *p* = .006), with levels decreasing slightly but remaining fairly stable over time (slope = −13.14, *p* = .376). Relative to other pediatric populations (Mishel, 1983; Perez et al., 2019), this class is consistent with persistently low levels of uncertainty and, thus, named “low stable.”

### Class differences

To examine predictors of class membership, multinomial logistic regressions with R3STEP were used. Sociodemographic (i.e., parent relation to child, parent age), family (i.e., family DSD history, previous children), and parent adjustment (i.e., anxious and depressive symptoms) predictor clusters did not significantly relate to class membership (*p*s > .05). The child cluster (i.e., gender of rearing, diagnosis type, and known/unknown diagnosis) emerged as significant for diagnosis type and known/unknown diagnosis. Results demonstrated that parents of children with a congenital adrenal hyperplasia diagnosis were more likely (*p* < .001) to be in the “low-stable” class compared to parents of children with other diagnoses (e.g., 5-alpha reductase deficiency, gonadal dysgenesis, androgen insensitivity syndrome). Additionally, parents of children with unknown diagnoses were more likely (*p* < .001) to be in the “low-stable” class compared to parents of children with known diagnoses. However, upon entering all predictors into one multivariate model, none emerged as significantly associated with class membership.

## Discussion

The current study takes a critical step to longitudinally examine parental illness uncertainty using person-centered approaches among a cohort of parents of children with atypical genital appearance due to a DSD. Multiple trajectories of illness uncertainty were indicated, partially supporting the proposed predictions and identifying a three-class model characterized by a moderate and stable class (“moderate stable”), a low and stable class (“low stable”), and a high then decreasing (“declining”) class. Notably, the majority of the sample (58.6%) fell in the moderate-stable class. In other words, from diagnosis to 12-month follow-up, most parents report consistently moderate levels of uncertainty surrounding their child’s diagnosis and treatment. Additionally, the smallest class (low stable; 11.1%) demonstrated fairly consistent, low ratings of uncertainty across the 12 months. Interestingly, these uncertainty ratings among the moderate-stable and low-stable classes are lower than previous reports among other pediatric populations (e.g., Mishel, 1983). These reported levels of illness uncertainty among parents of children with DSD may be an underestimate due to several factors, such as the size and nature of the sample captured. Specifically given that the majority of our study sample has greater access to resources, as reflected in engaging care at a multidisciplinary center, having socially privileged racial/ethnic identities (i.e., majority White), and having higher socioeconomic status, the present study may not capture the full range of families’ experiences. For those who are not able to travel and access care at multidisciplinary centers and experience systemic barriers and biases when accessing and navigating the healthcare system due to racial/ethnic identity and/or socioeconomic status, they may be more likely to rate higher levels of illness uncertainty based on findings from other pediatric populations (e.g., Guan et al., 2023). Additionally, the relative diagnostic certainty for the majority of families represented in this group (i.e., CAH diagnoses confirmed expediently with newborn screening results) may impact parents’ relative perceptions of uncertainty.

Although it might be expected that most parents’ uncertainty decreases as they adjust to the birth of a new child, a new diagnosis, and/or after surgical intervention, these two stable classes, which included most parents (69.7%), demonstrated quite consistent ratings of uncertainty over time. In fact, previous research suggests that parents’ overall distress decreases (Ellens et al., 2017) and satisfaction improves (Bernabé et al., 2018) following early surgical intervention. However, the present findings suggest stable levels of uncertainty despite surgical intervention after baseline for families who elected for surgery (*n *=* *52; 91.2%). Therefore, it may be that additional factors outside of genital appearance contribute to parental uncertainty. Specifically, parents may experience uncertainty surrounding their child’s gender identity (Ernst et al., 2018), how their child will be treated by peers, and issues around puberty and fertility (Crissman et al., 2011; Pasterski et al., 2014). It should be also taken into account that for the present sample, some of these developmental events may not have occurred yet and, thus, could be maintaining or contributing to parents’ uncertainty across these early time periods. As these developmental events occur, changes in trajectories of parents’ uncertainty may be observed. Further, parents’ uncertainty surrounding their child’s social development may not be adequately captured by the present measure that largely focuses on the child’s medical diagnosis, treatment, and prognosis. Future research using a more comprehensive measure of parents’ future-oriented concerns secondary to their child’s diagnosis may capture additional domains of uncertainty.

The declining class represented the second largest portion of the sample (30.3%), with these parents exhibiting initially moderate levels of uncertainty that ultimately decreased by 6 and 12 months to a level similar to that of the low-stable class. Given that parental uncertainty has been previously shown to decrease on average over time (Ellens et al., 2017; Roberts et al., 2020), it is likely that those in the declining class drive this overall downward turn. Although findings produced support for a possible two-class over the three-class model, the distinction provided between the declining and low-stable classes within the three-class model provides more nuanced and potentially clinically meaningful information. However, future research with larger and more diverse sample sizes may find support for additional trajectories.

The current results also identified possible diagnostic predictors of class membership. Given the varied diagnostic and medical evaluation procedures necessitated across DSD diagnoses and presentations, it is likely that parents’ uncertainty is impacted during this period. For example, as CAH is often identified at newborn evaluation and/or on newborn screening (Therrell et al., 2015), it is likely that parents of children with diagnoses other than CAH must undergo additional diagnostic procedures after birth before receiving a definitive diagnosis (Wisniewski et al., 2012) and, as a result, may experience greater uncertainty. Indeed, qualitative reports find that parents endorse increased uncertainty and anxiety during this waiting period and reductions in uncertainty following diagnostic testing (Gough et al., 2008). Additionally, the chronicity and management associated with different diagnoses and/or medical needs may impact parents’ uncertainty over time. For those children with non-CAH diagnoses, particularly some types of XY DSDs (e.g., gonadal dysgenesis), there is potential concern for tumor development if gonadal tissue is not removed (Kathrins & Kolon, 2016). For those parents, uncertainty related to possible future tumor development may have decreased following surgical intervention after the baseline timepoint (specifically, prophylactic removal of gonadal tissue to prevent tumor development). In contrast, CAH carries greater ongoing risk due to potential salt-wasting crises, as well as daily medication management tasks (Speiser et al., 2010) that remain stable over time, whether or not there is surgical intervention. Future research using larger samples across diagnoses is needed to better elucidate the impact of diagnostic procedures and medical intervention on parental uncertainty.

Of note, parent gender did not predict class membership as it did in previous research on trajectories of distress (Perez et al., 2021). However, this is consistent with previous reports that mothers and fathers demonstrate comparable levels of uncertainty (Delozier et al., 2019; Larasati et al., 2023).

### Strengths and limitations

The present study has several strengths. To our knowledge, it is the first study to examine trajectories of parental illness uncertainty longitudinally using person-centered analyses in this rare, understudied population. Further, the sample consists of a significant proportion of mother and father dyads, uniquely including data from fathers, who are largely underrepresented in the pediatric and DSD literature (Davis et al., 2019). Lastly, data was collected from multiple sites and, as a result, includes families across different regions of the United States with unique cultural nuances and access to resources.

Despite these strengths, certain limitations should be considered when interpreting findings. First, though analyses were bolstered to support examination of a smaller sample (e.g., full-information maximum likelihood estimation), the sample size was relatively small for these statistical approaches. Relatedly, the sample is largely homogenous in terms of race/ethnicity, socioeconomic status, educational background, and family structure. This homogeneity likely limits generalizability due to systemic biases that differentially impact families from marginalized backgrounds and communities. In fact, we were less likely to retain families from marginalized socioeconomic statuses and racial/ethnic identities, likely reflective of systemic biases and barriers to study participation. Of note, several marginalized racial/ethnic identity categories only contained one to very few participants. As such, participants were collapsed across BIPOC identities for statistical analysis. Individuals across racial/ethnic groups experience marginalization and systemic inequities in varied and complex ways. By collapsing across groups, these nuanced and varied experiences are likely not accounted for and limit the interpretability of systemic biases impacting study findings.

Additionally, families were recruited from multidisciplinary specialty treatment clinics and may not represent the experiences of families who receive care in other treatment settings. Also, most families in the present study opted for surgery; therefore, the experiences of families who choose to forgo elective surgery may differ from those captured here. Families were specifically recruited for children who presented with atypical genital appearance *without* additional organ malformations; thus, parental uncertainty may differ for those navigating a DSD diagnosis in addition to other medical risk factors. Lastly, as discussed previously, the parental illness uncertainty measure used does not directly capture parents’ uncertainties regarding gender identity and decisions around gender of rearing, which may not fully represent the nuance of uncertainty as it relates to diagnosis and treatment in this population. Future longitudinal research is needed with a more representative, diverse sample, and using more comprehensive measurements of parents’ appraisals validated for DSD.

### Clinical implications

It is critical to understand the nature of parents’ uncertainty over time, as this information can help inform clinical intervention. Parents of children with DSD who report greater illness uncertainty are more likely to also report greater anxious and depressive symptoms (Roberts et al., 2020). Additionally, parents who elect for surgical intervention and report greater preoperative uncertainty are more likely to endorse greater decisional regret later on surrounding their child’s surgery (Fisher et al., 2022). Further, among other pediatric populations, parental uncertainty and coping are linked to child uncertainty and coping (Baudino et al., 2019).

Although uncertainty is inherent in life and indeed while parenting and navigating chronic medical needs, psychosocial providers are poised to optimize positive coping and support overall family adjustment. Previous research has demonstrated efficacy for managing uncertainty using cognitive-behavioral intervention strategies such as informational support, emotion regulation, and self-management skill training (Guan et al., 2021; Wright et al., 2009), as well as acceptance and commitment therapy (Burke et al., 2014) and narrative therapy approaches (Eeltink & Duffy, 2004). When supporting families coping with a child’s diagnosis and uncertainty, it is imperative that providers use an integrative, socioecological family systems approach for conceptualization and intervention (Kazak et al., 2006), as those with DSD are often navigating sociocultural expectations surrounding sex and gender during an extremely sensitive period following birth.

## Conclusion

In sum, the present study contributes to a gap in the pediatric DSD literature by identifying patterns of parental illness uncertainty over time. Although average ratings of uncertainty decrease over time and are lower than that of other chronic illness populations, there are distinguishable classes of parents that demonstrate varying degrees of stability or decrease in uncertainty over the course of a year. These trajectories may be related to factors associated with different diagnostic testing and treatment experiences across families. Future studies are needed to evaluate how these trajectories might remain stable or change as children age and families navigate key developmental tasks and events. Moreover, adapting and validating evidence-based intervention strategies to address uncertainty in families of children with DSD can further support providers in promoting family adjustment and coping.

## Supplementary Material

jsae043_Supplementary_Data

## Data Availability

The data underlying this article cannot be shared publicly to maintain the privacy of individuals that participated in the study. The data will be shared on reasonable request to the corresponding author.
