# Supplementary material for: Trajectories of illness uncertainty among parents of children with atypical genital appearance due to differences of sex development
Source: J Pediatr Psychol. 2024 Jun 10;49(8):559–70. doi: 10.1093/jpepsy/jsae043 (PMC11335143; doi:10.1093/jpepsy/jsae043)
Supplement: jsae043_Supplementary_Data [file jsae043_supplementary_data.docx]

**Figure 1**

*Raw Score Plot*

******
